# Retraction: Therapeutic interventions impact brain function and promote post-traumatic growth in adults living with post-traumatic stress disorder: A systematic review and meta-analysis of functional magnetic resonance imaging studies

**DOI:** 10.3389/fpsyg.2024.1501838

**Published:** 2024-10-01

**Authors:** Frontiers Editorial Office

The journal retracts the February 09 2023 article cited above.

Following publication, concerns were raised regarding the scientific validity of the article. An investigation was conducted in accordance with Frontiers' policies.

It was found that the complaints were valid and that the article does not meet the standards of editorial and scientific soundness for Frontiers in Psychology; therefore, the article has been retracted.

This retraction was approved by the Chief Editors of Frontiers in Psychology and the Chief Executive Editor of Frontiers. The authors agree to this retraction.

Frontiers would like to thank Jenny Ann Rydberg for contacting the journal regarding the published article.

